# Enhancing Immersion in Virtual Reality–Based Advanced Life Support Training: Randomized Controlled Trial

**DOI:** 10.2196/68272

**Published:** 2025-02-14

**Authors:** Dilek Kitapcioglu, Mehmet Emin Aksoy, Arun Ekin Ozkan, Tuba Usseli, Dilan Cabuk Colak, Tugrul Torun

**Affiliations:** 1 Center of Advanced Simulation and Education Acibadem Mehmet Ali Aydinlar University Istanbul Turkey; 2 Department of Medical Education Medical Faculty Acibadem Mehmet Ali Aydinlar University Istanbul Turkey; 3 Institute of Biomedical Engineering Boğaziçi University Istanbul Turkey; 4 Department of Anesthesia Vocational School of Health Services Acibadem Mehmet Ali Aydinlar University Istanbul Turkey; 5 Department of Psychology Faculty of Humanities and Social Sciences Acibadem Mehmet Ali Aydinlar University Istanbul Turkey

**Keywords:** artificial intelligence, voice recognition, serious gaming, immersion, virtual reality

## Abstract

**Background:**

Serious game–based training modules are pivotal for simulation-based health care training. With advancements in artificial intelligence (AI) and natural language processing, voice command interfaces offer an intuitive alternative to traditional virtual reality (VR) controllers in VR applications.

**Objective:**

This study aims to compare AI-supported voice command interfaces and traditional VR controllers in terms of user performance, exam scores, presence, and confidence in advanced cardiac life support (ACLS) training.

**Methods:**

A total of 62 volunteer students from Acibadem Mehmet Ali Aydinlar University Vocational School for Anesthesiology, aged 20-22 years, participated in the study. All the participants completed a pretest consisting of 10 multiple-choice questions about ACLS. Following the pretest, participants were randomly divided into 2 groups: the voice command group (n=31) and the VR controller group (n=31). The voice command group members completed the VR-based ACLS serious game in training mode twice, using an AI-supported voice command as the game interface. The VR controller group members also completed the VR-based ACLS serious game in training mode twice, but they used VR controllers as the game interface. The participants completed a survey to assess their level of presence and confidence during gameplay. Following the survey, participants completed the exam module of the VR-based serious gaming module. At the final stage of the study, participants completed a posttest, which had the same content as the pretest. VR-based exam scores of the voice command and VR controller groups were compared using a 2-tailed, independent-samples *t* test, and linear regression analysis was conducted to examine the effect of presence and confidence rating.

**Results:**

Both groups showed an improvement in performance from pretest to posttest, with no significant difference in the magnitude of improvement between the 2 groups (*P*=.83). When comparing presence ratings, there was no significant difference between the voice command group (mean 5.18, SD 0.83) and VR controller group (mean 5.42, SD 0.75; *P*=.25). However, when comparing VR-based exam scores, the VR controller group (mean 80.47, SD 13.12) significantly outperformed the voice command group (mean 66.70, SD 21.65; *P*=.005), despite both groups having similar time allocations for the exam (voice command group: mean 18.59, SD 5.28 minutes and VR controller group: mean 17.3, SD 4.83 minutes). Confidence levels were similar between the groups (voice command group: mean 3.79, SD 0.77 and VR controller group: mean 3.60, SD 0.72), but the voice command group displayed a significant overconfidence bias (voice command group: mean 0.09, SD 0.24 and VR controller group: mean –0.09, SD 0.18; *P*=.002).

**Conclusions:**

VR-based ACLS training demonstrated effectiveness; however, the use of voice commands did not result in improved performance. Further research should explore ways to optimize AI’s role in education through VR.

**Trial Registration:**

ClinicalTrials.gov NCT06458452; https://clinicaltrials.gov/ct2/show/NCT06458452

## Introduction

Serious game–based training modules have become one of the training modalities for simulation-based health care training and have an important role in the training of health care professionals [1-5]. Training using serious gaming modules can be conducted through various platforms such as personal computers, tablets, virtual reality (VR), or mixed reality systems like augmented reality or augmented virtuality. The portability and increased affordability of VR systems in recent years have contributed to significant growth in VR-based learning. Providing immersive and interactive experiences of VR technology has revolutionized various fields including gaming, education, and training of health care professionals [1,6-8].

Enhanced student engagement makes VR and mixed reality modules particularly favored for health care training, such as advanced cardiac life support (ACLS) training, which aims to train health care professionals in the management of cardiopulmonary arrest. More than a million health care professionals participate in ACLS or advanced life support (ALS) courses globally each year [9]. Since these courses must be repeated at specific intervals, as mandated by local regulations or institutional requirements, software-based training modules and VR-based serious games will allow trainees to refresh their knowledge and skills anytime and anywhere [10].

VR-based training modules have used hand-held controllers for navigation and interaction with the virtual environment so far. In parallel to the advancements in artificial intelligence (AI) and natural language processing (NLP) potential for voice command interfaces, they offer an alternative method of control in VR applications by providing more intuitive and accessible means of interaction for users [11-15]. Voice commands, powered by NLP algorithms, enable users to navigate and manipulate virtual environments using natural language and have the potential to reduce the learning curve associated with VR controller inputs. Voice interfaces have also the potential to offer accessibility benefits for users with physical disabilities, for whom conventional controllers may be difficult to use [14].

Despite the potential advantages, using voice command systems in VR gaming has also its limitations like speech recognition accuracy, latency, the system’s ability to understand context-specific commands, problems caused by environmental noise, and the user’s accent or speech patterns [16,17].

There has been limited research comparing the user performance and user satisfaction of AI-supported voice command interfaces against traditional VR controllers [11,13,15]. This study aims to compare the 2 control methods using various metrics like task completion time, participants’ exam scores, level of confidence, and level of presence. As the level of presence gives insight into how deeply the learner is immersed in the game, while confidence ratings reveal how the learner perceives their own performance and decision-making, the level of presence and confidence ratings during gameplay were also compared [18-20]. The hypothesis of this study is that AI-supported voice commands have the potential to offer a superior user experience and user performance in VR gaming by providing a natural form of interaction. The research questions of the study are the following.

- How do participants’ pretest and posttest scores differ when using AI-supported voice commands versus traditional VR controllers?
- Do AI-supported voice command interfaces provide a superior user experience compared to traditional VR controllers in terms of performance using VR-based exam scores?
- How do confidence ratings vary between users of AI-supported voice command interfaces and traditional VR controllers during gameplay?
- How does the perceived level of presence differ between users of AI-supported voice commands and traditional VR controllers in VR gaming?

## Methods

### Recruitment

This study included 67 volunteer students from Acibadem Mehmet Ali Aydinlar University Vocational School for Anesthesiology. The participants, aged between 20-22 years, were in their fourth semester (spring semester 2023-2024) of the anesthesiology program. Five students declined to participate in this study. The remaining participants were randomly assigned to 2 groups: 31 students using VR controllers for game interaction (VR controller group) and 31 students using AI-supported voice recognition for game interaction (voice command group). The exclusion criteria for the study included prior ACLS training, a history of VR-induced motion sickness, and medical conditions such as vertigo attacks or using medications causing vertigo-like symptoms. Three participants from the voice command group were excluded for not meeting the inclusion criteria. The randomization was based on the participants’ university ID numbers, with odd numbers assigned to one group and even numbers to the other. The clinical trial registration identifier of the study is ClinicalTrials.gov NCT06458452.

### Ethical Considerations

This study was conducted in compliance with ethical guidelines for human subject research. The research protocol was reviewed and approved by the Scientific Ethical Committee of Acibadem Mehmet Ali Aydinlar University (ATADEK 2024-6). For the primary data collection, informed consent was obtained from all participants in accordance with the guidelines set forth by the scientific ethical committee of Acibadem Mehmet Ali Aydinlar University. The informed consent obtained for the primary study explicitly allowed for the deidentified data to be used in subsequent research, including secondary analyses, without requiring additional consent. This study was conducted with strict adherence to privacy and confidentiality standards to protect the data of all participants. The study data were deidentified prior to analysis, ensuring that no personally identifiable information was accessible during the research process. Any potential identifiers were removed or coded to maintain confidentiality throughout the study. Participation in the study was entirely voluntary, and no compensation was provided to the participants. All images included in the study and supplementary materials do not feature individual participant images or any identifying clues that could reveal the identity of the users.

### Study Flow

All the participants completed a pretest consisting of 10 multiple-choice questions about ACLS, which served to assess the participants’ initial knowledge level and whether the content was compatible with the latest version of ACLS (Multimedia Appendix 1) [21].

Following the pretest, participants were randomly divided into 2 groups: the voice command group (n=31) and the VR controller group (n=31). The voice command group members completed the VR-based ACLS serious game in training mode twice, using an AI-supported voice command as the game interface. The VR controller group members also completed the VR-based ACLS serious game in training mode twice, but they used VR controllers as the game interface. Although having volunteered for the study, 3 participants from the voice command group did not attend the study.

The participants from both groups completed the Turkish version of the Presence Questionnaire (PQ), a 7-point Likert scale, to estimate the level of presence during gameplay [22]. The participants rated their experience on a scale from 1=not at all to 7=completely by selecting the option that best reflects their experience. The PQ was first developed by Witmer and Singer [23] to subjectively measure the level of presence in 3D virtual environments. The scale was revised in a subsequent study conducted in 2005, which identified its factor structure as consisting of 4 factors [24]. The adaptation of the PQ to Turkish was conducted by Gokoglu and Cakiroglu [22] in 2019. The factor analysis of the Turkish version of the PQ found a 5-factor structure—involvement, sensory fidelity, adaptation or immersion, interaction, and interface quality—which was confirmed through a confirmatory factor analysis. The Turkish version of the questionnaire used in this study, along with its translated English version, are provided in Multimedia Appendices 2 and 3.

In the involvement factor of the scale, there are items for evaluating a situation obtained because of focusing the individual’s attention and mental potential consistently or meaningfully on relevant stimuli, activities, or events. For the sensory fidelity factor, items related to perceiving the VR scenario visually, auditorily, and haptically were included. The adaptation or immersion factor includes items related to being included in and interacting with a continuous flow of experiences and stimuli with a sense of being surrounded, while the interface quality factor includes items to evaluate the effect of visual and control interfaces in the VR experience. Finally, in the interaction factor, items related to interaction between individuals and the virtual environment were included. The adapted scale was found to be valid and reliable with a Cronbach α=.84 and applicable with its 29 items under 5 factors [22]. Participants’ presence score was calculated as the mean of their responses to all items in the scale.

Following the survey, participants were asked to indicate how they believed they would perform in the upcoming exam on a scale of 1 to 5, where 1=very bad and 5=very good, to assess confidence in future performance. After confidence assessments, participants completed the exam module of the VR-based serious gaming module, which evaluates the technical and nontechnical of the participants. The voice command group used a voice interface, while the VR controller group used VR controllers to complete the exam. At the final stage of the study, participants completed a posttest, which had the same content as the pretest.

### Serious Gaming Module Used for the Study

The software used in this study is a VR-based ALS serious game that has been developed in accordance with the ACLS guidelines European Resuscitation Council and American Heart Association [21,25]. The development team worked closely with clinicians to ensure compliance with these guidelines as well as crisis resource management criteria [26-28].

This serious game works with a learning management system and keeps user credentials as well as game results in a shared database with the help of a Learning Record Store [29-31]. The interactions of the players with the virtual world are tracked using a 3D visualization engine and experience application programming interface calls are created accordingly [30,32]. These calls are sent to the Learning Record Store servers with the help of a unity extension library that uses HTTP protocol [33]. This library also includes security measures and authentication methods included with HTTP protocols required for the safekeeping of user data.

The VR-based ALS serious game includes an AI-driven voice recognition feature that uses NLP algorithms of a service called Wit.ai [34]. Wit.ai is an open-source service that has existing Unity plug-ins that facilitate the implementation process. Through this service, the sentences of the user are sent to wit.ai servers, processed into game commands according to predefined keywords, and returned to determine the action taken by the user.

The serious gaming module offers 2 language options: English and Turkish. Participants were given the choice between the 2 options. As all volunteers preferred Turkish during gameplay, both the VR controller group and voice command groups selected Turkish as the game language. Since the voice command group also chose Turkish, the AI-driven voice recognition was based on the Turkish model. There are 2 stages in this serious game: beginner training and VR-based exam. These stages can be played by either voice command or using the controller of the VR headset. The simulated environment consists of a virtual hospital room and equipment to immerse the user as much as possible in this experience.

**Figure 1:**
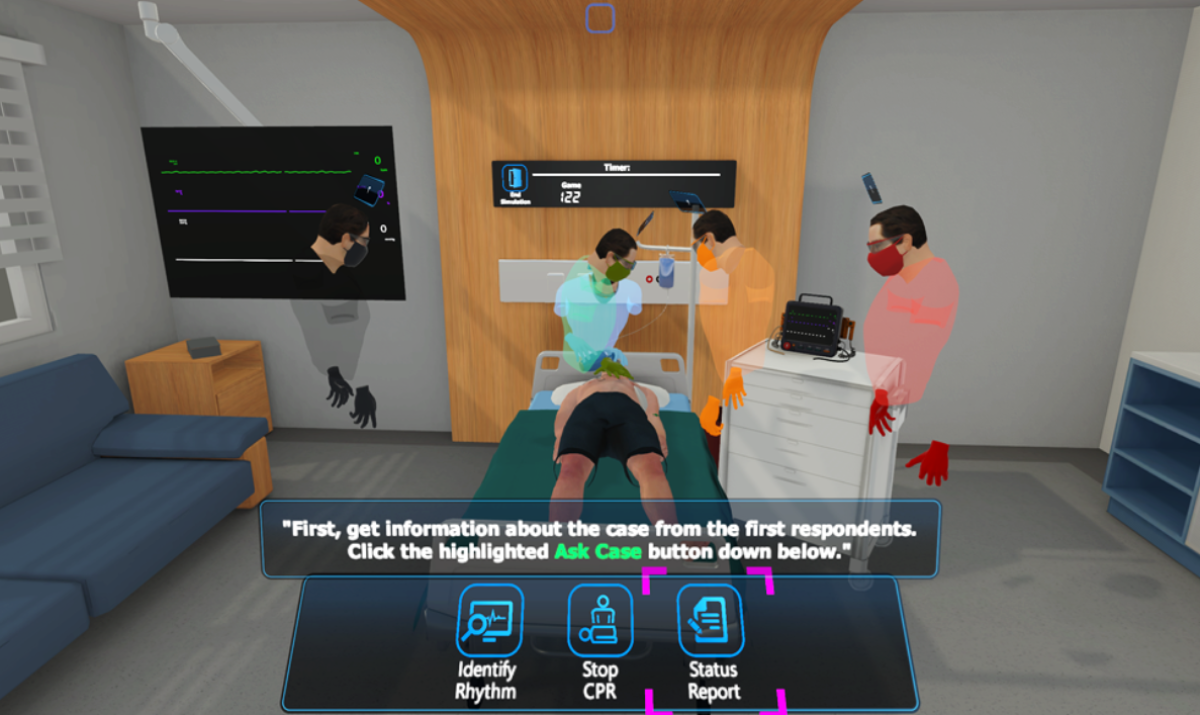
Screen capture of the laser pointer–controlled mode of the advanced cardiac life support serious game. CPR: cardiopulmonary resuscitation.

**Figure 2:**
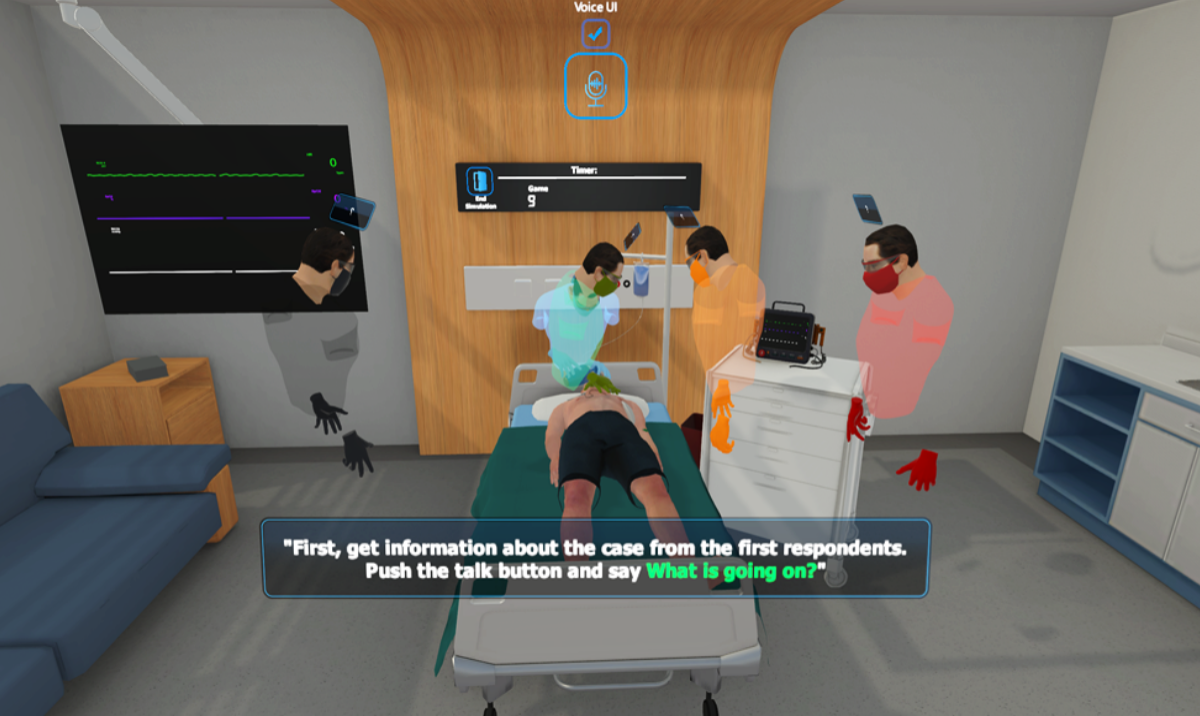
Screen capture of the voice-control mode of the advanced cardiac life support serious game.

The beginner training stage is directed toward users who are willing to familiarize themselves with the ACLS algorithm and the VR environment. In this stage, the user is completely guided from the beginning to the end using visual and audio cues. Which objects to interact with or which voice commands to say are given to the users? Examples of guidance can be seen in Figure 1 and Figure 2 respectively. There are also no timing constraints in this mode which removes the stress element and allows an inexperienced user to focus on learning the algorithm [8].

The exam stage is designed for users who are confident in their abilities after spending some time in the basic training stage. Each action of the user is scored according to its accuracy, order, and timing. Each action taken by the user is graded based on its timing, order, and accuracy. The final score of the VR-based exam, out of 100 points, is derived from 2 categories: 70% (70/100) from ACLS assessments and 30% (30/100) from crisis resource management performance. The scores of the exam mode were stored and could be accessed afterward.

### Data Analysis

Data from the comparison of pretest and posttest scores, VR-based exam scores, confidence ratings, presence ratings, and time spent in training mode were evaluated to compare the 2 groups. Additionally, the relationship between VR-based exam scores and time spent in training was assessed. Given the presence of multiple variables, a variety of statistical tests were used for data analyses based on the nature of the addressed question. To compare the performance change from pretest to posttest across the voice command and VR controller groups, a 2×2 mixed ANOVA was used. VR-based exam scores of voice command and VR controller groups were compared using 2-tailed, Welch independent-sample *t* test as the homogeneity of variances assumption was not met. Confidence ratings of the voice command and VR controller groups were compared using a Shapiro-Wilk test as the data were not normally distributed. Confidence bias was assessed using a 2-tailed, 1-sample *t* test. Presence scores of the groups and time spent in training mode across groups were compared using a 2-tailed, Student independent-sample *t* test.

Prior to analysis, the dataset was screened for outliers across all variables. No outliers were detected; thus, no data points were removed. The central tendency of the data was described using the mean. Effect sizes were reported as Cohen *d* (0.2=small, 0.5=medium, and 0.8=large) for *t* tests and η²*P* (0.01=small, 0.06=medium, and 0.14=large) for ANOVA.

Each reported analyses were deemed appropriate based on data distribution and variance homogeneity. Data distribution was checked using the Shapiro-Wilk test of normality and homogeneity of variances was assessed by performing the Levene test of equality of variances. Details regarding the selection of the statistical analyses used can be found in Multimedia Appendix 4.

Data was analyzed using JASP (Version 0.19.3, JASP Team), an open-source software for statistical analysis. The figures were created using JASP.

## Results

### Overview

**Figure 3:**
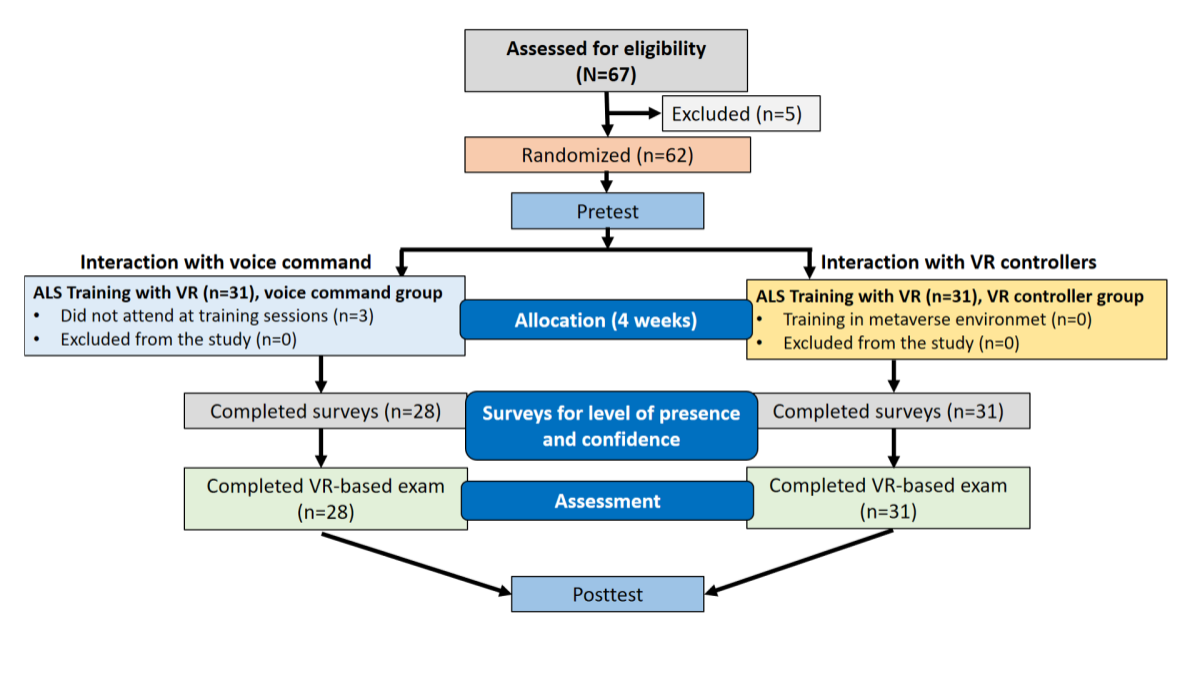
CONSORT (Consolidated Standards of Reporting Trials) flow diagram of the study. ALS: advanced life support; VR: virtual reality.

The following analysis was conducted with 59 participants, as shown in Figure 3.

### Change of Scores from Pretest to Posttest

**Figure 4:**
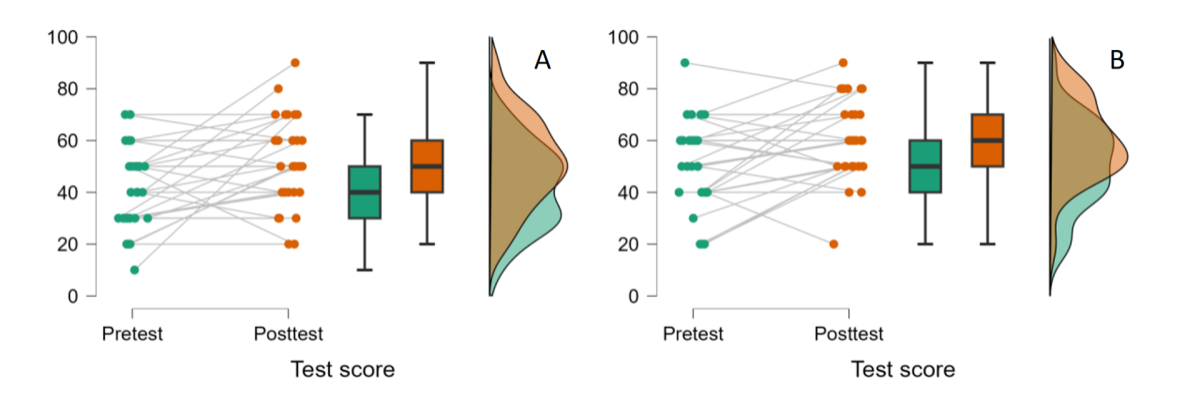
Pretest and posttest scores for (A) VR controller group and (B) voice command group, respectively. Dots indicate each participant’s test score. The boxes indicate IQRs; the line in the middle of the boxes indicates the median. Lines between dots connect each participant’s pretest and posttest scores. VR: virtual reality.

To reveal whether performance change from the pretest to the posttest is different between groups, a 2 (group: voice command group vs VR controller group) × 2 (test: pretest vs posttest) mixed ANOVA where the first factor is the between-subjects variable and the second is the within-subjects variable was conducted. Levene test showed that the assumption of homogeneity of variances across groups was met for both pretest (*P*=.85) and posttest scores (*P*=.54). Furthermore, a Shapiro-Wilk test for normality showed that pretest scores of the voice command group (*P*=.14) and VR controller group (*P*=.15) were normally distributed. Similarly, posttest scores of both groups were also normally distributed (*P_voice command group_*=.09 and *P_VR controller group_* =.43). Considering that the within-subjects variable has only 2 levels, a test for sphericity assumption was not required. Hence, the analysis was conducted without any correction as all assumptions were met. The results showed that the voice command group performed better than the VR controller group in general, (*F*_1,57_=6.43; *P*=.01; *n^2^p*=0.10). Moreover, performance was higher in the posttest than in the pretest for both groups (*F*_1,57_=15.69; *P*<.001; *n^2^p*=0.22). This increase in performance from pretest to posttest, however, was not different for voice command and VR controller groups, as the interaction between group and test was not significant (*F*_1,57_= 0.05; *P*=.83; *n^2^p*=8.18×10^–4^). These findings indicate that while the voice command group had a higher performance in general, both groups showed a performance increment from pretest to posttest, the increase in performance was not higher in one group than the other (Figure 4).

### Comparison of VR-Based Exam Scores

**Figure 5:**
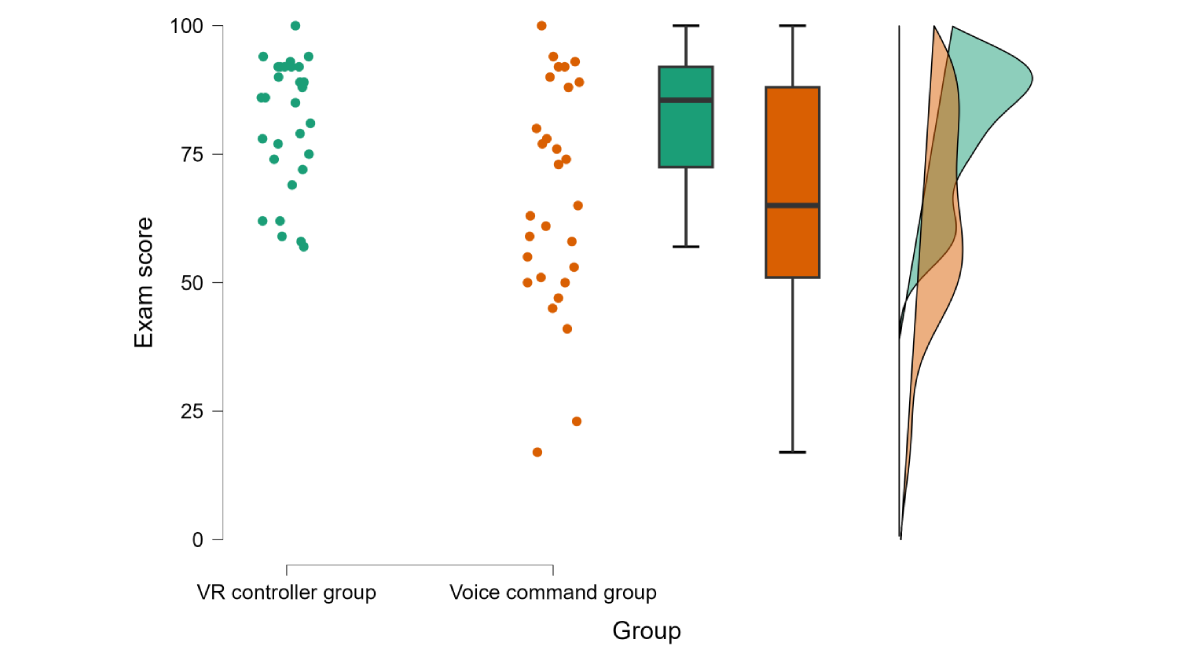
Scores of VR-based exam mode for VR controller and voice command groups. Dots indicate each participant’s exam score. The boxes indicate IQR; the line in the middle indicates the median. VR: virtual reality.

**Figure 6:**
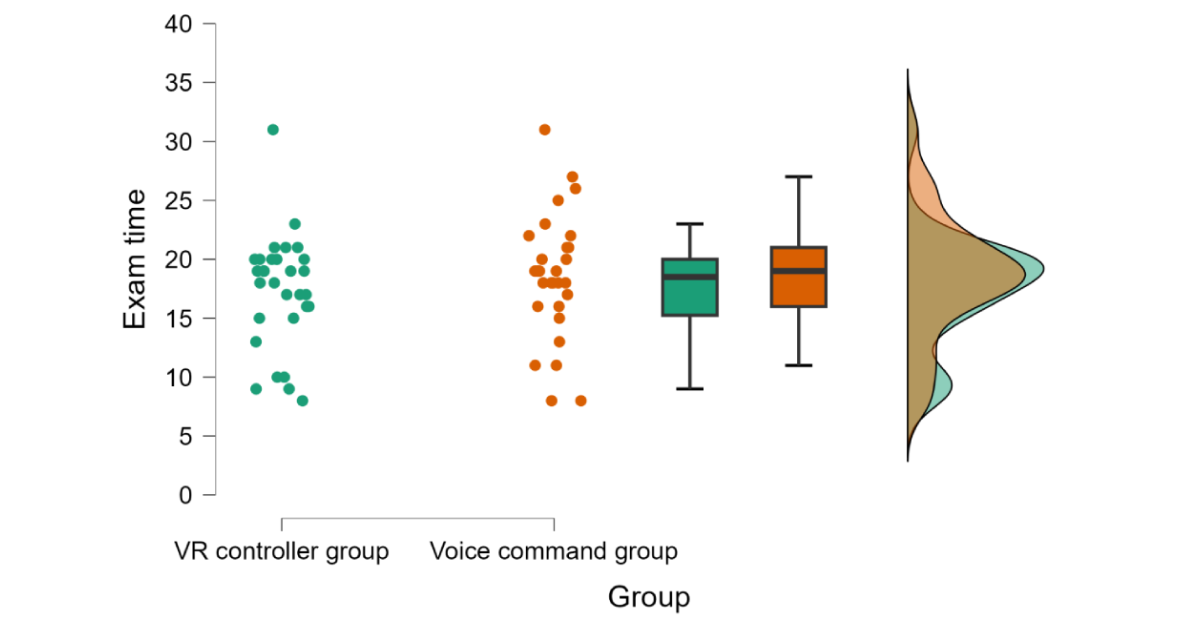
Time spent on the advanced training stage for VR controller and voice command groups in minutes. Dots indicate each participant’s time. The boxes indicate IQRs; the line in the middle indicates the median. VR: virtual reality.

Crucial to the purpose of the study, an independent-sample, 2-tailed Welch *t* test showed that the VR controller group’s performance in the VR-based exam (mean 80.47, SD 13.12) was higher than the voice command group’s performance (mean 66.70, SD 21.65; t_45.83_=2.94; *P*=.005; *d*=0.77, 95% CI 0.23-1.30; Figure 5). Moreover, both groups spent a similar amount of time in the exam (t_57_=–0.95; *P*=.35; *d*=–0.25, 95% CI –0.76 to 0.27; voice command group: mean 18.59*_,_* SD 5.28 and VR controller group: mean 17.33, SD 4.83; Figure 6).

### Confidence Ratings

Results revealed that there was no significant difference between groups’ confidence ratings (W=392.00; *P*=.49, *r_b_*=.15, 95% CI –0.38 to 0.20; voice command group: mean 3.79, SD 0.77; VR controller group: mean 3.60, SD 0.72).

**Figure 7:**
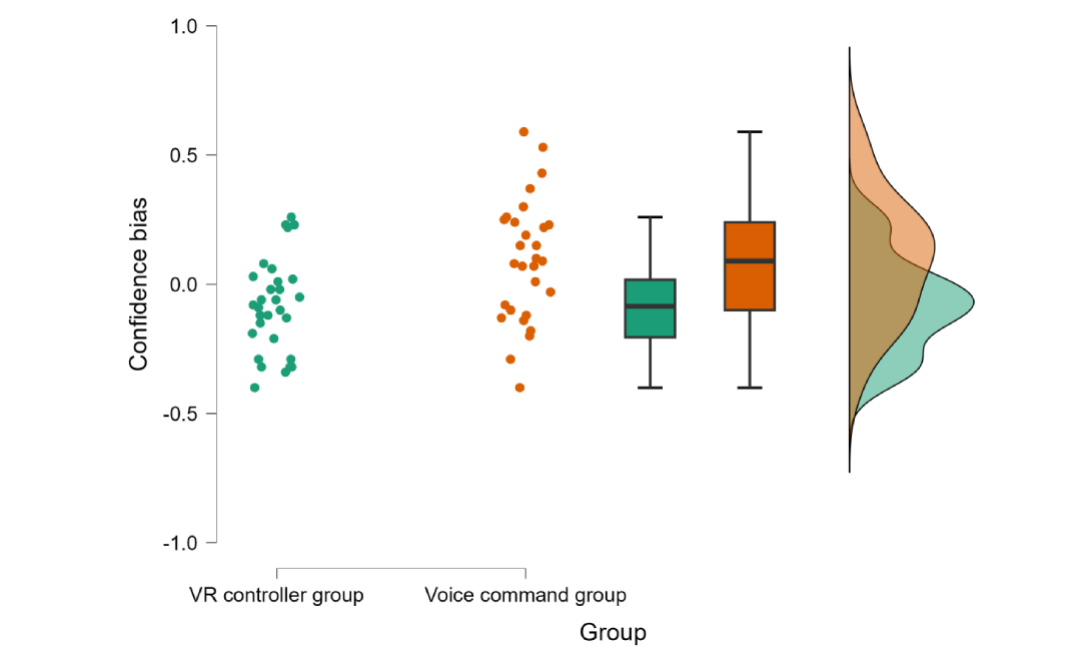
Confidence bias for VR controller and voice command groups. Dots indicate each participant’s confidence bias. The boxes indicate IQRs; the line in the middle indicates the median. VR: virtual reality.

In general, there is a positive relationship between performance and confidence levels, such that higher performance is accompanied by higher confidence levels [18,35]. Therefore, given the lower performance of the voice command group, it can be expected that their confidence ratings would also be lower. The absence of this expected difference points to a confidence bias in groups voice command group, that is, a mismatch between confidence and performance [36]. To see if this is the case, confidence bias for each participant was calculated by scaling their confidence ratings and advanced training performance over 1 and subtracting the scaled performance from the scaled confidence rating, a commonly used method for confidence bias quantification [37]. A 2-tailed, independent-sample *t* test showed a significant difference, with the voice command group’s performance and confidence difference (mean 0.09, SD 0.24) being bigger than the VR controller group’s performance and confidence difference (mean –0.09, SD 0.18; t_57_=–3.21; *P*=.002; *d*=–0.83, 95 CI –1.37 to –0.30; Figure 7). To clarify the possible source of the group differences in confidence bias, a 2-tailed, 1-sample *t* test was conducted for each group. The results showed that the mean confidence bias of the VR controller group was significantly lower than 0, indicating an underconfidence bias (t_29_=–2.56; *P*=.02; *d*=–0.47), and the mean confidence bias of the voice command group was significantly higher than 0, indicating an overconfidence bias (t_28_=2.08; *P*=.047; *d*=0.39).

### Comparison of Presence Ratings

The 2-tailed, independent-sample *t* test showed that the presence ratings did not differ across groups (t_57_=1.16; *P*=.25; *d*=0.30, 95% CI –0.21 to 0.81; voice command group: mean 5.18, SD 0.83; VR controller group: mean 5.42, SD 0.75).

## Discussion

### Principal Findings

This study explored the potential of AI-driven voice control in VR-based ACLS training. The findings revealed that both groups improved from pretest to posttest and the VR controller group achieved higher scores in the VR-based exam compared to the voice command group. This suggests that traditional controllers may currently provide a more reliable interaction method in VR environments. Analyses confirmed that the data followed a normal distribution and satisfied the assumption of equal variances, with the exceptions being exam scores and confidence ratings, for which appropriate corrections were applied to address the assumption violations. These corrections, along with the reported effect sizes demonstrating medium to large effects across the majority of significant findings, underscored the robustness of the results and the adequacy of the sample size.

Despite the potential advantages, using voice command systems in VR gaming has also its limitations in speech recognition accuracy, latency, the system’s ability to understand context-specific commands, problems caused by environmental noise, and the user’s accent or speech patterns. Voice command systems in VR gaming present both promising advantages and notable limitations [17,38-41]. While these systems offer an intuitive, hands-free interaction method, their effectiveness can be hindered by factors like speech recognition accuracy, latency issues, context-specific command understanding, environmental noise, user’s accent, and speech patterns. Errors in recognizing words or phrases can disrupt gameplay and may lead to reduced immersion. Delays in system response to voice commands can significantly impact the flow of VR experiences, particularly in fast-paced gaming scenarios where real-time interaction is crucial. Research shows that even minor delays can break the sense of presence and reduce overall engagement. The ability of a system to interpret context-specific commands remains a challenge. For instance, players might use commands with implied meanings or colloquial expressions that the system struggles to process, affecting functionality and the seamlessness of gameplay. Research highlights that background noise significantly impacts voice recognition performance. In noisy environments, players might need to repeat commands, which can be both disruptive and exhausting, diminishing the immersive qualities of VR gaming. Studies also show that accents, dialects, and individual speech patterns affect recognition accuracy. Systems trained on limited datasets may struggle with diverse user inputs, leading to unequal performance across different user demographics [17,38-41].

These literature data underscore the potential problems of AI-guided voice recognition in terms of accuracy and performance in different languages [42,43]. Since Turkish was selected as the language for AI-driven voice recognition in this study, there were similar difficulties due to the challenges of voice recognition, which caused participants in the voice command group to lose time and achieve lower scores compared to the VR controller group.

The level of presence did not significantly differ between the 2 groups, which contrasts with prior expectations that voice control would enhance immersion [18,19]. The level of presence may be hindered by problems in word recognition and delays in system response [17,38-41].

Confidence ratings were comparable across groups; however, an interesting finding was the presence of an overconfidence bias in the voice command group and an underconfidence bias in the VR controller group. This suggests that the mode of interaction can influence learners’ self-perception of their performance. Aligning perceived competence with actual performance remains a critical goal for optimizing educational outcomes. While they are related, confidence bias and task performance are dissociable [36], making it important to examine both to better understand the learning process. Although both groups reported comparable confidence levels, the voice command group exhibited a significant overconfidence bias while the VR controller group exhibited a significant under-confidence bias. This discrepancy highlights the importance of aligning perceived competence with actual performance outcomes in educational settings [44] and indicates that the means of education can differentially affect this alignment.

Although current limitations in language recognition pose challenges, ongoing advancements in AI and NLP technologies may close these gaps and provide more effective solutions in the future. These limitations indicate that while voice command systems present innovative possibilities in VR gaming, developers need to address these issues to enhance the user experience and ensure accessibility for a wider audience.

### Limitations

The first limitation of the study is the potential for selection bias, as participants were recruited from a single institution, which limits the generalizability of the results to other educational settings or demographics. The second limitation is the use of AI-driven voice recognition, which is prone to issues such as speech accuracy, environmental noise, and user-specific factors like accent, all of which could impact user experience and performance. A third limitation is the lack of longitudinal follow-up assessments to evaluate the long-term retention of skills, which is essential for assessing the effectiveness of VR-based training methods.

### Conclusions

The results of this study highlight the effectiveness of VR-based training for ACLS. Although voice commands did not enhance the exam performance, level of presence, and confidence level of the participants in this study, voice commands showed a potential promise as an innovative interaction method. Addressing challenges such as language-specific recognition accuracy could significantly improve their application in VR-based educational modules.

Future research should focus on optimizing AI-driven interfaces and exploring their role in fostering learner immersion and confidence. Enhancing these aspects could lead to improved educational outcomes and better preparation for real-world scenarios in health care.

## Appendix

Multimedia Appendix 1 Advanced cardiac life support (ACLS) pre- and posttest exams.

Multimedia Appendix 2 The Turkish version of the questionnaire.

Multimedia Appendix 3 Translated version of the questionnaire.

Multimedia Appendix 4 Statistical data.

Multimedia Appendix 5 CONSORT-EHEALTH checklist.

## Abbreviations

ACLS: advanced cardiac life support
AI: artificial intelligence
ALS: advanced life support
NLP: natural language processing
PQ: Presence Questionnaire
VR: virtual reality

## Data Availability

The datasets generated or analyzed during this study, as well as the serious gaming module, are available from the corresponding author upon request.
