# Supplementary material for: Enhancing Immersion in Virtual Reality–Based Advanced Life Support Training: Randomized Controlled Trial
Source: JMIR Serious Games. 2025 Feb 14;13:e68272. doi: 10.2196/68272 (PMC11888007; doi:10.2196/68272)
Supplement: Multimedia Appendix 1 [file games_v13i1e68272_app1.pdf]

Pre-Test and Post-Test Questions

ADVANCED LIFE SUPPORT IN ADULTS: PRE-TEST AND POST-TEST QUESTIONS

Case: A 60-year-old male patient develops postoperative sudden cardiopulmonary arrest in a ward bed. The ward physician and two nurses initiate basic life support after issuing a code blue.

Question 1: As the code blue team leader, what steps should you follow in the correct order upon arriving at the scene?

- **I.** Check the rhythm
- **II.** Stop chest compressions
- **III.** Assess the patient's condition
- **IV.** Delegate tasks
- **V.** Check the pulse

A. I-II-III-IV-V
B. III-IV-II-V-I
C. V-IV-II-I-III
D. II-IV-V-I-II
E. III-IV-V-I-II

Question 2: Which of the following is/are shockable rhythm(s)?

- **I.** Atrial fibrillation
- **II.** Asystole
- **III.** Ventricular fibrillation
- **IV.** Pulseless electrical activity
- **V.** Ventricular tachycardia
- **VI.** Pulseless ventricular tachycardia

A. IV
B. V
C. I-V
D. III-VI
E. II-IV

Question 3: Which of the following is/are non-shockable rhythm(s)?

- **I.** Atrial fibrillation

- **II.** Asystole
- **III.** Ventricular fibrillation
- **IV.** Pulseless electrical activity
- **V.** Ventricular tachycardia
- **VI.** Pulseless ventricular tachycardia

A. IV
B. V
C. I-V
D. III-VI
E. II-IV

Question 4: What energy level should be delivered using a biphasic defibrillator for a shockable rhythm?

A. 100 J
B. 120 J
C. 200 J
D. 250 J
E. 350 J

Question 5: When should medication administration occur in a non-shockable rhythm?

A. Immediately after taking over the patient
B. Before delegating tasks
C. Immediately after the initial assessment
D. After changing the person performing chest compressions
E. At the most appropriate time after the first rhythm analysis

---

Question 6: When should medication administration occur in a shockable rhythm?

A. Immediately after rhythm analysis
B. After starting chest compressions following the first shock
C. Before starting chest compressions after the second shock
D. Before starting chest compressions after the third shock
E. After starting chest compressions following the fourth shock

---

Question 7: What medication and dosage should be administered for a non-shockable rhythm?

A. 1 mg Adrenaline + 300 mg Amiodarone (every 3-5 minutes)
B. 1 mg Adrenaline + 150 mg Amiodarone
C. 1 mg Adrenaline (every 3-5 minutes) + 300 mg Amiodarone
D. 1 mg Adrenaline (every 3-5 minutes)
E. 300 mg Amiodarone (every 3-5 minutes)

Question 8: What medication and dosage should be administered for a shockable rhythm?

A. 1 mg Adrenaline + 300 mg Amiodarone (every 3-5 minutes)
B. 1 mg Adrenaline + 150 mg Amiodarone
C. 1 mg Adrenaline (every 3-5 minutes) + 300 mg Amiodarone
D. 1 mg Adrenaline (every 3-5 minutes)
E. 150 mg Amiodarone (every 3-5 minutes)

Question 9: When should intubation and capnography be performed/used?

A. Immediately after taking over the patient
B. Before delegating tasks
C. Immediately after the initial assessment
D. After changing the person performing chest compressions
E. At the most appropriate time after the second assessment

Question 10: How often should the patient be assessed and the person performing chest compressions be changed?

A. After 1 cycle (30 compressions/2 ventilations)
B. After 2 cycles (30 compressions/2 ventilations)
C. After 3 cycles (30 compressions/2 ventilations)
D. After 4 cycles (30 compressions/2 ventilations)
E. After 5 cycles (30 compressions/2 ventilations)
