# Supplementary material for: Enhancing Immersion in Virtual Reality–Based Advanced Life Support Training: Randomized Controlled Trial
Source: JMIR Serious Games. 2025 Feb 14;13:e68272. doi: 10.2196/68272 (PMC11888007; doi:10.2196/68272)
Supplement: Multimedia Appendix 2 [file games_v13i1e68272_app2.pdf]

BULUNUŞLUK HİSSİ ÖLÇEĞİ

(***Turkish Version of the Presence Questionnaire***)

Aşağıda yer alan her bir ifadeyi dikkatlice okuyunuz ve gerçekleştirdiğiniz sanal gerçeklik deneyimini göz önünde bulundurarak soruları yanıtlayınız. Size en yakın gelen seçeneği işaretleyerek deneyiminizi 1 (hiç) ile 7 (tamamen) arasında derecelendiriniz.

(Carefully read each statement below and answer the questions considering your virtual reality experience. Rate your experience on a scale from 1 (not at all) to 7 (completely) by selecting the option that best reflects your experience.)

	1	2	3	4	5	6	7
1. Olayları ne kadar kontrol edebildiniz?							
2. Başlattığınız (veya gerçekleştirdiğiniz) eylemlere ortam ne kadar tepki verdi.							
3. Ortamla etkileşimleriniz ne kadar doğal görünüyordu?							
4. Ortamın görsel yönleri sizi ne kadar içine aldı?							
5. Ortamdaki sesler sizi ne kadar içine aldı?							
6. Ortam içerisinde hareketin kontrol edildiği sistem ne kadar doğaldı?							
7. Ortamda hareket eden nesnelerin sizde uyandırdığı his ne kadar inandırıcıydı?							
8. Sanal ortamdaki deneyimleriniz gerçek dünyadaki deneyimlerinizle ne kadar tutarlı görünüyordu?							
9. Gerçekleştirdiğiniz eylemlere karşılık olarak bir sonraki adımda ne olacağını tahmin edebildiniz mi?							
10. Gözlüğü kullanarak ortamı ne kadar aktif bir şekilde inceleyebildiniz veya araştırabildiniz?							
11. Sesleri ne kadar tanıyabildiniz?							
12. Seslerin geldiği yeri ne kadar belirleyebildiniz?							
13. Dokunma aracını kullanarak sanal ortamı ne kadar aktif olarak inceleyebildiniz veya araştırabildiniz?							
14. Sanal ortam içerisindeki etrafta gezinme hissi ne kadar inandırıcıydı?							
15. Nesneleri ne kadar yakından inceleyebildiniz?							
16. Nesneleri farklı bakış açılarından ne kadar inceleyebildiniz?							
17. Sanal ortamdaki nesneleri ne kadar hareket ettirebildiniz veya yönlendirebildiniz?							
18. Sanal ortam deneyimine ne kadar dahil oldunuz?							
19. Ortamdaki hareketleriniz ile hareketlerinizin beklenen sonuçları arasında ne kadar gecikme yaşadınız?							
20. Sanal ortam deneyimine ne kadar çabuk uyum sağladınız?							
21. Yaşadığınız deneyim sonrasında sanal ortamda hareket etme ve etkileşime girme konusunda kendinizi ne kadar yeterli hissettiniz?							
22. Verilen görevleri veya gerekli etkinlikleri yerine getirirken, gözlüğün görüntü kalitesi sizi ne kadar engelledi veya dikkatinizi dağıttı?							
23. Kontrol cihazları, verilen görevlerin veya diğer etkinliklerin yerine getirilmesini ne kadar engelledi?							

	1	2	3	4	5	6	7
24. Ortamdaki görev veya etkinlikleri yerine getirmek için kullanılan sistemlerden çok, verilen görevlere veya etkinliklere ne kadar konsantre olabildiniz?							
25. Duyularınız bu deneyimi ne kadar yoğun yaşadı?							
26. Nesneleri fiziksel etkileşim yoluyla tanımak (bir nesneye dokunmak, bir yüzeyin üzerinde yürümek veya bir duvar veya nesneye çarpmak) ne kadar kolay oldu?							
27. Sanal ortam deneyimi sırasında ortama veya göreve tamamen odaklandığınızı hissettiğiniz anlar oldu mu?							
28. Sanal ortamla etkileşim kurmak için kullanılan kontrol cihazlarına ne kadar kolay uyum sağladınız?							
29. Sanal ortamda farklı duyularla sağlanan bilgiler (örneğin görme, duyma, dokunma) tutarlı mıydı?							
