# Supplementary material for: Enhancing Immersion in Virtual Reality–Based Advanced Life Support Training: Randomized Controlled Trial
Source: JMIR Serious Games. 2025 Feb 14;13:e68272. doi: 10.2196/68272 (PMC11888007; doi:10.2196/68272)
Supplement: Multimedia Appendix 3 [file games_v13i1e68272_app3.pdf]

ENGLISH TRANSLATION of the PRESENCE QUESTIONNAIRE

Carefully read each statement below and answer the questions considering your virtual reality experience. Rate your experience on a scale from 1 (not at all) to 7 (completely) by selecting the option that best reflects your experience.

	1	2	3	4	5	6	7
1. How much were you able to control events?							
2. How responsive was the environment to actions that you initiated (or performed)?							
3. How natural did your interactions with the environment seem?							
4. How much did the visual aspects of the environment involve you?							
5. How much did the auditory aspects of the environment involve you?							
6. How natural was the mechanism which controlled movement through the environment?							
7. How compelling was your sense of objects moving through space?							
8. How much did your experiences in the virtual environment seem consistent with your real world experiences?							
9. Were you able to anticipate what would happen next in response to the actions that you performed?							
10. How completely were you able to actively survey or search the environment using vision?							
11. How well could you identify sounds?							
12. How well could you localize sounds?							
13. How well could you actively survey or search the virtual environment using touch?							
14. How compelling was your sense of moving around inside the virtual environment?							
15. How closely were you able to examine objects?							
16. How well could you examine objects from multiple viewpoints?							
17. How well could you move or manipulate objects in the virtual environment?							
18. How involved were you in the virtual environment experience?							
19. How much delay did you experience between your actions and expected outcomes?							
20. How quickly did you adjust to the virtual environment experience?							
21. How proficient in moving and interacting with the virtual environment did you feel at the end of the experience?							
22. How much did the visual display quality interfere or distract you from performing assigned tasks or required activities?							
23. How much did the control devices interfere with the performance of assigned tasks or with other activities?							
24. How well could you concentrate on the assigned tasks or required activities rather than on the mechanisms used to perform those tasks or activities?							

	1	2	3	4	5	6	7
25. How completely were your senses engaged in this experience?							
26. How easy was it to identify objects through physical interaction; like touching an object, walking over a surface, or bumping into a wall or object?							
27. Were there moments during the virtual environment experience when you felt completely focused on the task or environment?							
28. How easily did you adjust to the control devices used to interact with the virtual environment?							
29. Was the information provided through different senses in the virtual environment (e.g., vision, hearing, touch) consistent?							
