# Supplementary material for: Enhancing Immersion in Virtual Reality–Based Advanced Life Support Training: Randomized Controlled Trial
Source: JMIR Serious Games. 2025 Feb 14;13:e68272. doi: 10.2196/68272 (PMC11888007; doi:10.2196/68272)
Supplement: Multimedia Appendix 4 [file games_v13i1e68272_app4.pdf]

Change of Scores from Pretest and to Posttest Scores

Levene's test showed that assumption of homogeneity of variances across groups was met for both pretest (*F* (1,57) = .04, *P* = .85) and posttest scores (*F* (1, 57) = .38, *P* = .54). Furthermore, a Shapiro-Wilk test for normality showed that pretest scores of both VG group (*W* = .95, *P* = .14) and CG group (*W* = .95, *P* = .15) were normally distributed. Similarly, posttest scores of both groups were also normally distributed (W_{VG} = .94, P = .09; W_{CG} = .97, *P* = .43). Considering that the within-subjects variable has only 2 levels, a test for sphericity assumption was not required. Hence, a 2x2 mixed ANOVA was conducted without any correction as all assumptions were met.

Comparison of VR-based Exam Scores

A Shapiro-Wilk test for normality showed a deviation from normality for the CG group (W = .90, P = .006) but not for the VG group (W = .95, P = .23). Additionally, Levene's test indicated that the assumption of homogeneity of variances was not met (*F* (1, 57) = 8.30, *P* = .006). As a result, it was deemed appropriate to use a Welch's independent samples t-test.

Comparison of Exam Time

A Shapiro-Wilk test for normality showed a deviation from normality for the CG group (*W* = .91, *P* = .02) but not for the VG group (*W* = .97, *P* = .50). Assumption of homogeniety of variances was not violated as shown by Levene's test, *F* (1, 57) = .08, *P* = .78). Therefore, a Student's independent samples t-test was deemed appropriate.

Comparison of Confidence Ratings+

While Levene's test showed that the assumption of homogeneity of variances was met, *F* (1, 57) = .19, *P* = .66, Shapiro-Wilk test showed distribution of data was normal for neither the VG group (*W* = .79, *P* < .001) nor the CG group (W = .83, P < .001). Considering that both groups had a significant deviation from normality, Mann-Whitney test was used as a non-parametric alternative for Student's t-test.

Comparison of Presence Ratings

The normality of the data was assessed using the Shapiro-Wilk test, which indicated that the data were normally distributed for both the VG group (*W* = .96, *P* = .30) and the CG group (*W* = .94, *P* = .07). Additionally, Levene's test confirmed that the assumption of homogeneity of variances was met (*F* (1, 57) = 1.33, *P* = .26). As both assumption were satisfied, a Student's t-test was deemed appropriate for the analysis.

Comparison of Confidence Bias

Shapiro-Wilk test showed that the distribution of confidence bias was normal for both the VG group (*W* = .99, *P* = .98) and the CG group (*W* = .96, *P* = .26). Furthermore, Levene's test revealed that assumption of homogeneity of variances was met, *F* (1,57) = 2.00, *P* = .16. As all assumptions were met, a Student's independent samples t-test -was employed.
